# Electronic Tongue for Brand Uniformity Control: A Case Study of Apulian Red Wines Recognition and Defects Evaluation †

**DOI:** 10.3390/s18082584

**Published:** 2018-08-07

**Authors:** Larisa Lvova, Irina Yaroshenko, Dmitry Kirsanov, Corrado Di Natale, Roberto Paolesse, Andrey Legin

**Affiliations:** 1 Laboratory of Artificial Sensory Systems, ITMO University, 197101 St. Petersburg, Russia; irina.s.yaroshenko@gmail.com (I.Y.); d.kirsanov@gmail.com (D.K.); dinatale@uniroma2.it (C.D.N.); roberto.paolesse@uniroma2.it (R.P.); andrey.legin@gmail.com (A.L.); 2 Department of Chemical Sciences and Technology, University ‘Tor Vergata’, 00133 Rome, Italy; 3 Institute of Chemistry, St. Petersburg State University, 198504 St. Petersburg, Russia

**Keywords:** potentiometric e-Tongue, wine brand uniformity control, defect compounds analysis

## Abstract

The potentiometric electronic tongue system has been tested as a potential analytical tool for brand uniformity control of monoculture Apulian red wines (Primitivo and Negroamaro). The sensor array was composed of eight porphyrin coatings obtained by electrochemical polymerization process and was employed for both wines discrimination and quantitative detection of wine defect compounds: “off-odour” 3-(methylthio)-propanol; isoamyl alcohol fusel oil; benzaldehyde (marker of the yeast activity) and acetic acid (marker of vinegar formation). PLS-DA applied to Electronic tongue output data has permitted a correct discrimination of more than 70% of analysed wines in respect to the original brand affiliation. Satisfactory PLS1 predictions were obtained in real wine samples; with R^2^ = 0.989 for isoamyl alcohol and R^2^ = 0.732 for acetic acid. Moreover; the possibility to distinguish wine samples on the base of permitted levels of fault compounds content was shown.

## 1. Introduction 

The well recognizable organoleptic properties of food and drink products are essential to maintain the brand uniformity and play a major role in consumer choices and preferences. In wine, the combination of taste and aroma but also of bouquet, appearance and mouth feel results in the unique appearance of a determined brand [1]. The wine composition is a complex mixture of hundreds of compounds, arising from original grapes and/or formed during alcoholic fermentation [2]. The balance of wine composition can be significantly influenced even by the minor concentration variation of only one taste responsible component. Such a variation may completely change or drastically damage the overall wine perception, influence negatively a brand uniformity and became a significant problem for consumers. Moreover, since the wine fraud remains nowadays an essential issue worldwide [3], the accurate control of product authenticity and adulteration detection are extremely important in the wine industry. According to the literature [3,4]: “Wine adulteration can be committed through dilution with water, addition of alcohol or other substances and blending with, or replacement by, wine of a lesser quality” and also “… wine fraud can be committed through misinformation about the wine, such as mislabelling.”

An application of wet chemistry procedures [5] and several instrumental techniques [6,7,8,9,10], such as high-performance liquid chromatography (HPLC), gas chromatography combined with mass spectrometry (GC-MS), Fourier Transform Infrared (FTIR) spectroscopy, stable isotope ratio analysis through nuclear magnetic resonance (NMR), isotope ratio mass spectrometry (IRMS), are the most employed analytical methods in wine detection technology. Being quite sophisticated, these techniques require a costly equipment, long time of analysis and trained personnel involvement. Such analyses give information on individual wine components but they cannot provide the global assessment of wine flavour or quality, that makes impossible the most wine frauds detection. 

An involvement of professional wine tasting panels remains until nowadays indispensable in the wine authentication analysis. However, recent studies show that the human perception of wine organoleptic characteristics is very subjective and it is very difficult to identify wines in blind tastings. Even being very skilled, human panellists may perform only few assessments per day, due to the saturation of tongue receptors, and, as reported in [11], among the provided judgments only half of the panellists were consistent with their previous evaluations of the same wine.

The possibility to substitute human sensory panels with artificial olfaction and taste sensory systems, such as Electronic Nose (e-Nose), Electronic Tongue (e-Tongue), has been actively investigated over the last two decades [12,13,14] and was especially explored for the wine industry [15,16,17,18,19]. Although artificial sensory systems cannot provide information about the analysed sample without preliminary calibration and/or additional analytical determination, they may provide a cheap, rapid and not tedious possibility for either the classification of samples of different quality, or the quantitative determination of several parameters. 

Among different types of electrochemical sensors, potentiometric sensors and sensor arrays have been actively employed for wine analysis [20,21], since potentiometry provides the simple measurement procedure and does not change irreversibly the composition of analysed samples, which is important, for instance, during the wine fermentation assessment. Most of the previously reported sensors were prepared by incorporation of membrane-active components inside inert polymeric matrix and then deposited on the transducer (via simple adhesion or either chemical linking) to the transducer surface. 

Various options for ligand grafting, such as photopolymerization [22], covalent attachment to polymer [23,24], electropolymerization of monomers bearing electrochemically active unit or their co-polymerization with intrinsically conductive polymers, such as polypyrrole, polyaniline and so forth [25,26,27,28,29,30] have been exploited previously in the literature. Among them, the last method permits to obtain a stable sensing film chemically attached to the transducer surface, to increase significantly the sensor lifetime and to avoid a ligand leaching, which could be a serious problem for the alcoholic environment analysis, as wine is. Porphyrins, thanks to their rich chemistry and particular chelating ability, appear very promising ligands for the development of potentiometric chemical sensors and multisensory systems for wine analysis, including wine authentication, taste evaluation and faults detection [31,32,33]. 

In the present work, an e-Tongue system based on porphyrin electropolymers, obtained by electrodeposition of n-alkyl-(1-pyrrole) phenyl-substituted porphyrins or aminophenyl substituted porphyrin polymeric coatings, was employed for the authenticity assessment of 24 samples of Apulian wines produced by different cantinas and made of 100% Primitivo (10 wines) or Negroamaro (14 wines) grape varieties. Moreover, the correct discrimination of authentic Negroamaro wine and wine contaminated with several fault compounds was performed by the developed e-Tongue system. The possibility to detect in analysed Apulian wines the defects related to the presence of so-called wine “off-odour” compound 3-(methylthio)-propanol (methionol), isoamyl alcohol fusel oil, the marker of the yeast activity benzaldehyde and acetic acid as wine acidification marker was also investigated; the satisfactory results were obtained for isoamyl alcohol and acetic acid in particular.

## 2. Experimental

### 2.1. Reagents

Tartaric acid, acetic acid, ethyl alcohol, isobutyl alcohol, isoamyl alcohol, 3-(methylthio)-propanol and benzaldehyde were purchased from Sigma-Aldrich (Milan, Italy). The solvents acetonitrile (ACN) and dichloromethane (CH_2_Cl_2_) were from Sigma-Aldrich. 5,10,15-tris(4-aminophenyl)-20-phenylporphyrinates of Cu(II) and Co(II) (**1** and **4**), mono 5-(4-aminophenyl)-10,15,20-phenylporphyrinates of FeCl(III) and Mn(III) (**2** and **3**), 5-{4-[5-(pyrrol-1-yl) pentyloxy] phenyl},10,15,20-(triphenyl) porphyrin of Co(II) (**5**), 5-{4-[5-pyrrol-1-yl)decyloxy]phenyl},10,15,20-(triphenyl) porphyrin of Co(II) (**6**), 5,10-di4-[5-(pyrrol-1-yl) phenyl],10,20-(di-mesityl) porphyrin (**7**), 5-{4-[5-(pyrrol-1-yl)-decyloxy]phenyl},10,15,20-(trimesityl) porphyrin (**8**) were synthesized in our laboratories according to the previously reported procedures [28,34,35] and fully characterized by NMR and UV-Visible spectroscopy. All other chemicals were of analytical grade and were used without further purification. For electrochemical measurements, the analytical grade solvents, electrolyte salts and tetrabutylammonium perchlorate supporting electrolyte salt (TBAClO_4_, Fluka, Buchs, Switzerland) were used. Solutions for potentiometric and optical evaluations were prepared with distilled water. 

### 2.2. Sensors Preparation and Evaluation 

**Figure 1:**
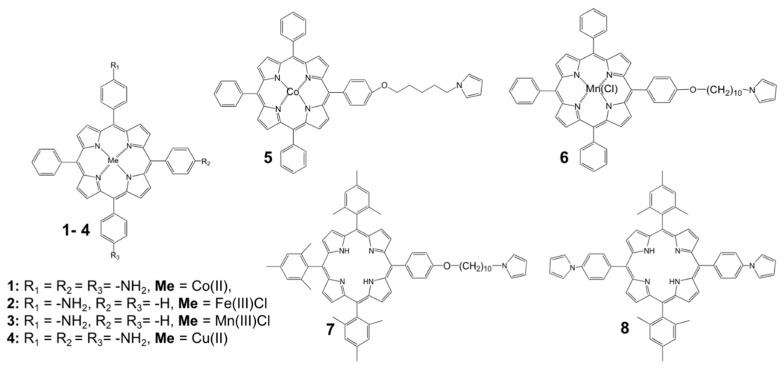
Chemical structures of porphyrin monomers employed in e-Tongue array.

The e-Tongue array was composed by 8 potentiometric chemical sensors obtained by electrodeposition of n-alkyl-(1-pyrrole) phenyl- and aminopenyl- substituted porphyrin polymeric coatings on flat Pt working electrodes surface (3 mm in diameter). The properties of utilized porphyrin electropolymer sensing films were studied in our previous works [28,29]. The films were formed via cyclic voltammetry technique, carried out in a three-electrode cell versus SCE reference and Pt wire counter electrodes, with AMEL 7050 (AMEL, Milan, Italy) potentiostat for 20 cycles, with 100 mV/s potential scan rate from the 1 mmol/L monomer solutions in CH_2_Cl_2_ and 0.1 M TBAClO_4_ as background electrolyte. Prior of the electropolymerization process, Pt WE surface was buffed with alumina slurries, cleaned in ultrasonic bath, rinsed with methanol and dried on air. After preparation, the sensors were soaked in 0.01 mol/L NaCl solution for 24 h and then tested versus ORION (model 90-02) reference electrode. The chemical structures of the employed porphyrin monomers are given in Figure 1. The compositions and electropolymerization conditions of the sensing materials employed in the e-Tongue are listed in Table 1. 

**Figure 2:**
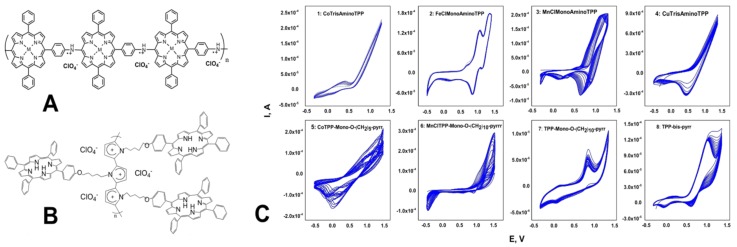
Schematic presentation of PANI (**A**) and PPy (**B**) porphyrin-substituted electropolymers formation; (**C**) CV curves of oxidative polymerization of compounds **1**–**8** on PT WE by repeated potential scans at 100 mV/s.

The typical CV curves for electropolymer depositions are shown in Figure 2. In a case of compounds **1**–**4**, a polyaniline (PANI) backbone with incorporated porphyrin units was formed on Pt WE surface, Figure 2A For the pyrrole-substituted porphyrin monomers **5**–**8**, during the electropolymerization at high oxidative potentials the polypyrrole (PPy) backbone bearing side-linked active porphyrin units was formed on Pt WE through the 2,5-positions of pyrrole, Figure 2B For both PANI and PPy porphyrin-substituted polymers, the perchlorate ions of the supporting electrolyte, TBAClO_4_, served as anionic dopants in order to maintain the polymer electroneutrality. As can be seen in Figure 2C, the formation of an almost isolating polymeric film on PT WE was registered for compounds **2**, **4** and **6**, since their the voltammogram profiles did not change during all 20 cycles of electropolymerization; while in other cases (see films **1**, **3**, **5**, **7** and **8**) the oxidative current was growing with increase of film thickness, indicating formation of conductive film on WE surface. The morphology of obtained polymeric coatings was characterized with AFM and SEM techniques [28,33]. The obtained films had an average thickness in a range from 0.1 to 1 μm [28] and the film morphology was strongly dependent on the monomer structure as it is reported in [33].

The potentiometric responses of the sensors have been studied in solutions of several salts, in a concentration range of 10^−7^–10^−1^ M in buffer background (pH 5.5 to 8.6), or in distilled water (with simultaneous pH control), as described in detail in [28,29]. Sensor potentials were measured versus double junction SCE reference electrode (AMEL, Milan, Italy) and recorded using high-impedance 8-channel potentiometer LiquiLab (Ecosens, Rome, Italy). An artificial wine solution was employed to mimic the real wine matrix, while testing e-Tongue response toward selected wine fault compounds. The artificial wine solution had the following composition: 5 g/L tartaric acid, 123 g/L ethyl alcohol, 0.3 g/L isobutyl alcohol, 0.06 g/L isoamyl alcohol, pH = 3.2. The calculated amounts of wine fault compounds were added in known volume of artificial wine solution prior the first measurement; every compound was tested in 4 different concentrations: C_s_-standard concentration, C_a_-alarm concentration, C_d1_-defect concentration 1, C_d2_-defect concentration 2. In total 16 calibration solutions were prepared. All the measurements were performed in triplicate and the final data matrix had a dimension: 4 compounds × 4 concentrations × 3 replicas × 8 sensors = 384 points. Between the measurements the solutions were stored in sealed containers with limited headspace.

### 2.3. Wine Samples

**Figure 3:**
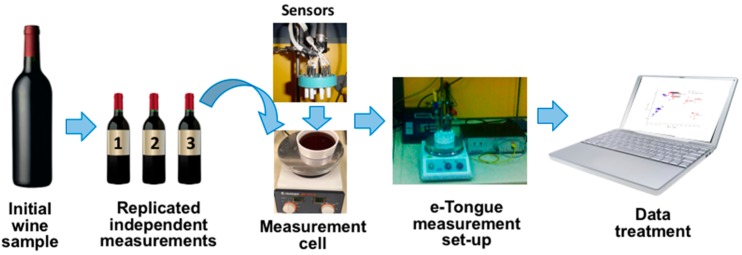
Schematic presentation of the employed measurement set-up.

The 24 samples of monovarietal Italian red wines produced in the Apulia region and bottled in 2007 by different cantinas were evaluated by e-Tongue array in order to confirm wines authenticity and reveal possible adulterations. Among them, 10 wine samples were made of 100% Primitivo and 14 wine samples were made of 100% Negroamaro grapes varieties correspondingly, Table 2. All wines were measured immediately after opening the bottles, without any sample pre-treatment, 3 times in a random order. e-Tongue array was first conditioned in distilled water and then in the artificial wine solution before and after measurement in every wine sample. Sensors were preserved in 0.01 M NaCl solution between consecutive measurements days. The sketch of the experimental set-up and e-Tongue image are given in Figure 3. 

### 2.4. Data Treatment

Principle component analysis (PCA) and Soft Independent Modelling by Class Analogy (SIMCA) were applied for wines identification and supervised classification respectively. Partial Least Regression (PLS1) method was used in order to correlate e-Tongue output with known amounts of wine defect compounds in calibration solutions. PLS Discriminant analysis, PLS-DA was employed for wines discrimination. Validation was performed using one-leave-out procedure. The RMSEP (Root Mean Square Error of Prediction) and correlation coefficient, R^2^, of predicted versus measured correlation line was used to evaluate the efficiency of applied regression model. Data treatment was performed with a commercial Unscrambler (v. 9.1, 2004, CAMO PROCESS AS, Oslo, Norway) software.

## 3. Results and Discussion

### 3.1. Electronic Tongue Tests in Artificial Wine Doped with Faults Compounds

The porphyrin electropolymers for e-Tongue have been chosen thanks to their particular binding properties when interacting with metal cations (for free base-porphyrins) or several anions (for porphyrin metallic complexes). Moreover, the enhanced cross-sensitivity of porphyrin electropolymers was shown recently and was one of the main criteria for the compounds **1**–**8** selection [28,29,31]. 

No tests on sensors sensitivity towards wine defect compounds were performed previously; for this reason, as a first task of the present work we have evaluated the e-Tongue array response toward several compounds responsible for the wine organoleptic faults: methionol (ME), isoamyl alcohol (IA), benzaldehyde (BA) and acetic acid (AA) in a wide range of concentrations (from maximum permitted or alarm levels in wine to the amounts in several times higher than the olfactory or taste threshold) [1]. The artificial wine solution was employed as background in order to mimic the real wine composition and to obtain as much reliable results, as possible. The amounts of wine defect compounds in artificial wine calibration solutions and in Negroamaro wine (NA14) used as real wine sample with adulterations are listed in Table 3.

ME arises as a reduction “off-odour” defect attributable to heavy sulphur compounds (as methionine), present in the must and providing an unpleasant odour of cooked cabbage. The ME formation occurs via methionine deamination and decarboxylation, followed by methional aldehyde formation, which is then enzymatically reduced to ME. ME perception threshold is 1.2 mg/L and higher concentrations are considered as a wine defect. IA is also called “fusel oil” and it is formed by yeast metabolism during alcoholic fermentation process from sugars and from amino acids (Erlich mechanism). IA is the major higher alcohol found in wines (more than 50%) and its concentration in wine has been reported in the range of 90 to 292 mg/L. IA may influence aromatic wine profile and hence its amount must be controlled. BA is the marker of the yeast activity in wine, it arises from enzymatic oxidation of benzyl alcohol by yeasts (especially *Schizosaccharomyces japponicus*). The typical BA concentration in wine varies significantly, usually being 0.015 mg/L but it may rich up to 6 mg/L; while olfactory threshold is 1.5–3.5 mg/L. BA provides to wine aroma and taste of bitter almonds; BA concentrations higher than 3 mg/L are considered as a wine defect. AA in wine is often referred to as volatile acidity (VA) or vinegar taint and it can originate from many wine spoilage yeasts and bacteria and forms either as by-product of fermentation, or due to the spoilage of finished wine by acetic acid bacteria, *Acetobacter* and *Gluconobacter*. Further during the esterification with ethanol, AA produces ethyl acetate and together this fault compounds give to wine a smell of vinegar, paint thinner and nail polish remover. The typical concentration of acetic acid in wine is in the range of 100–1150 mg/L, the sensory threshold for acetic acid is 700 mg/L; in concentrations greater than 1.2–1.3 g/L it becomes to be unpleasant.

The examples of potentiometric e-Tongue array response in artificial wine solutions doped with different concentrations of wine fault compounds are given in Figure S1. It can be seen from the Figure S1 the clear differences in sensors responses upon the concentration variation mainly of benzaldehyde, acetic acid and isoamyl alcohol. In fact, PLS1 regression method applied for e-Tongue calibration has shown a good correlation between the amount of added defect compound and potentiometric response of array with the correlation coefficients R^2^ of 0.925, 0.852 and 0.821 for BA, IA and AA respectively in all the tested range of concentration. These results indicate the utility of the developed potentiometric e-Tongue based on polymeric coating obtained from pyrrole- and aminophenyl-substituted porphyrin monomers **1**–**8** for adulterations assessment in artificial matrix of close to the wine composition.

### 3.2. Primitivo and Negroamaro Wines Assessment with Electronic Tongue 

Primitivo and Negroamaro are typical grape varieties of Apulia region of Italy. These grapes have a prevalent bitter taste and are generally employed for blending but they can also be used to produce monocultivar wines, which require ageing in order to receive a balanced, well recognized test. During the aging process, several aroma compounds arise in wines mainly from the grapes itself, from oak barrels or as the fermentation products. An enhanced concentration of these compounds may result in wine organoleptic faults. The wine samples listed in Table 2 were previously tested by SPE/GC–MS analysis and more than 50 volatile compounds were correctly identified (for Primitivo wines), 36 of them were present from 0.1 to 100 mg/L range, whereas the leftover 15 were present in not quantifiable traces [36]. Moreover, in the same paper an Electronic nose device based on eight Si-based MOX microsensors (Silsens SA, Neuchâtel, Switzerland, MSGS 4001-3005-3004-3003-3007 series) and chemometric PCA method were employed to distinguish between the two wine types.

**Figure 4:**
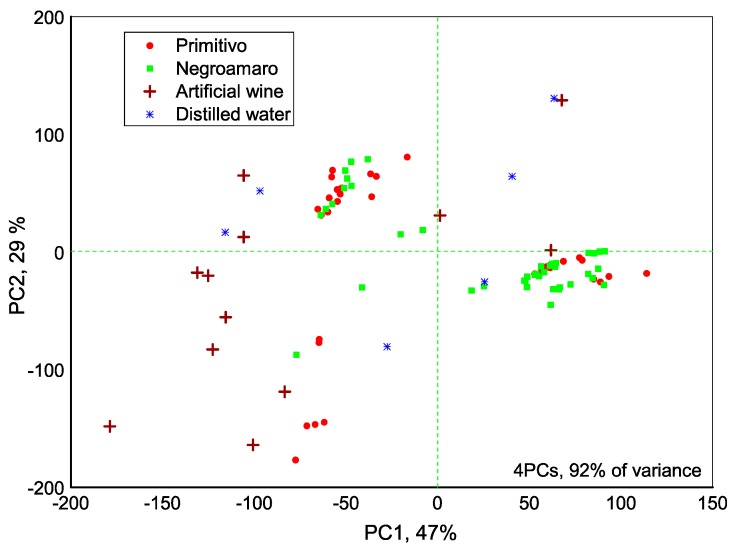
PCA score plot of e-Tongue data dispersion.

With an aim to distinguish Primitivo from Negroamaro wines and assure the brand uniformity by means of the developed e-Tongue, we have first evaluated the dispersion of sensor array data obtained in all tested samples with PCA method. A clear separation of artificial wine used for sensors conditioning before and after every measurement, distilled water used to clean sensors and real wines was observed, Figure 4. The 92% of the total variance was explained for 4 PCs and the highest influence (highest loadings) on samples discrimination was found for sensors **1**, **3**, **5** and **8** (see Table 1 for sensing material description).

**Figure 5:**
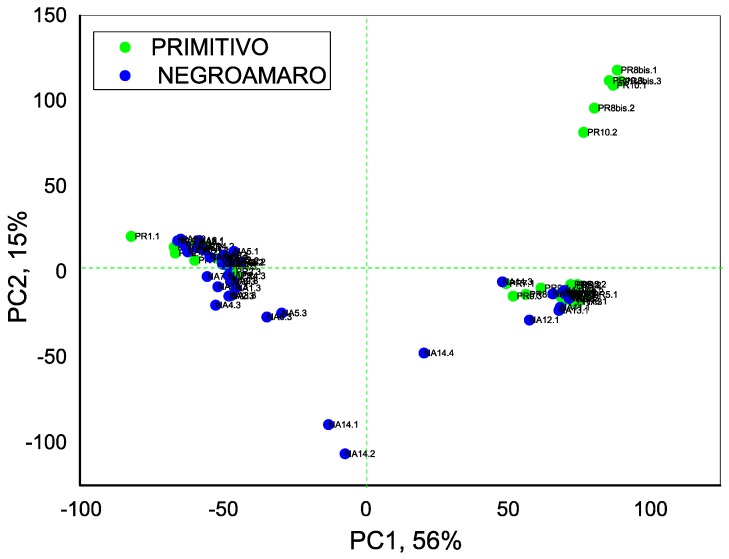
PCA-DA score plot of Primitivo and Negroamaro wines discrimination.

We have then passed to study the discrimination among real wines samples, 14 Negroamaro wines and 10 Primitivo wines. PLS-DA method was used for classification purposes, assigning to each wine type a separate class number. Due to the small data set (24 wine samples measured in triplicate by 8 sensors, 576 points in total) one-leave-out cross-validation of calculated model was employed. The obtained results are given in Figure 5 and Table 4. Three Negroamaro and four Primitivo wines were misclassified, thus resulting in correct discrimination of 71% of two authentic monovarietal Apulian wines by means of developed e-Tongue system.

**Figure 6:**
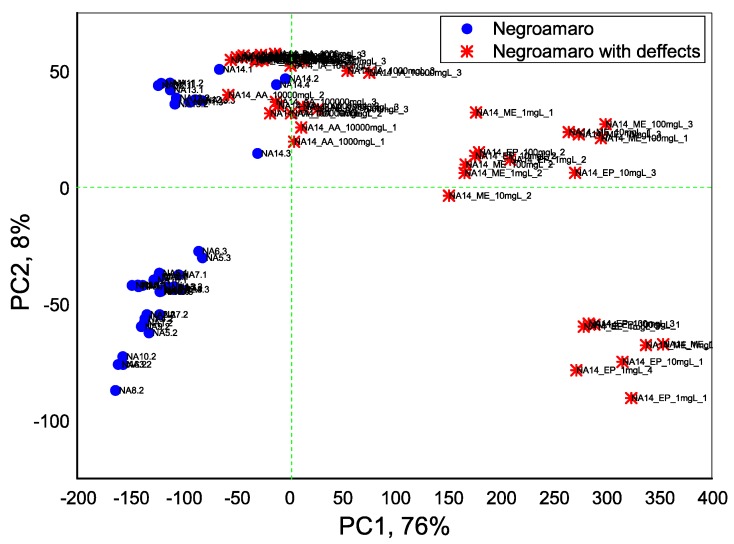
PCA-DA discrimination result among Negroamaro wines.

Inspired by the obtained result, we have performed an assessment of real Negroamaro wines, in order to identify the possible presence of defect compounds in the threshold, alarm and/or defect amounts. On the Figure S2 the potentiometric responses of e-Tongue array in the Negroamaro wine NA14, doped with the different concentrations of defect BA, AA, IA and ME compounds are shown. The significant difference in sensor array response can be noted for different samples. On the Figure 6 the PLS-DA classification of 29 Negroamaro wines (15 samples with added fault compounds and 14 authentic wines) is presented. The first two PCs represent 84% of total system variance and the clear discrimination among original Negroamaro wines and wines with adulterations was obtained. Besides, as it is demonstrated in Table 5, only one wine sample was misclassified and the correct classification of 97% of Negroamaro wines was obtained, thus demonstrating the utility of porphyrin electropolymers—based e-tongue for red wines defects detection.

Then the analysis of real Negroamaro wine samples was done in order to identify the presence of added defect compounds in the threshold, alarm and/or defect amounts. The adequate predictive power of e-Tongue was found for IA and AA, R^2^cal = 0.994 (R^2^val = 0.732, 4PCs) and R^2^cal = 0.999 (R^2^val = 0.989, 3PCs) correspondingly, while the lower correlation coefficients were received for other wine fault compounds. 

**Figure 7:**
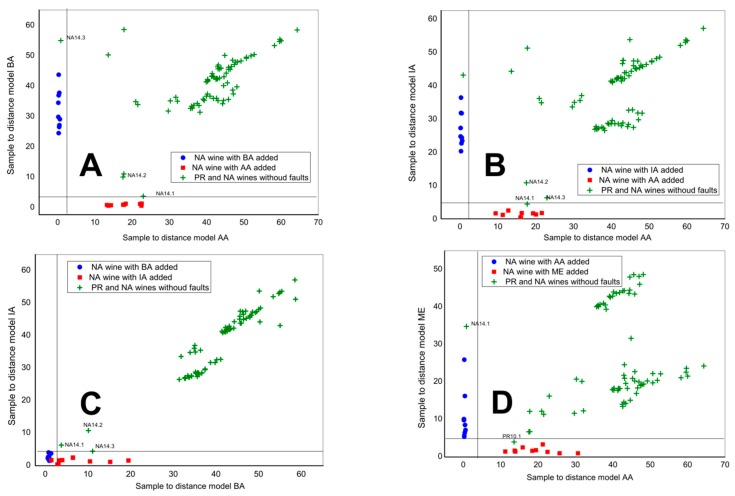
SIMCA classification Coomans plot of Apulian wines (green points) in respect to adulterations: (**A**) BA (blue points) and AA (red points); (**B**) IA (blue points) and AA (red points); (**C**) BA (blue points) and IA (red points); (**D**) AA (blue points) and ME (red points).

Finally, we have focused on the possible Primitivo and Negroamaro wines classification in relation to four fault compounds, BA, ME, IA and AA, tested in two-component SIMCA models. Negroamaro wine samples with added fault compounds in three different concentrations (corresponding to permitted amount, alarm and defect content) were used to create the models along the Coomans plot axis. Almost all Apulian wines were all classified as non-containing fault compounds in concentrations higher than the maximum permitted amount, Figure 7.

## 4. Conclusions

In this paper, we have reported a potentiometric e-Tongue based on polymeric coatings obtained from pyrrole- and aminophenyl-substituted porphyrins for Apulian red wines recognition and defects qualitative evaluation. The results obtained indicate a potential utility of the developed e-Tongue system as an effective tool of wine brand uniformity control, permitting an inexpensive, rapid and accurate monitoring of several fault compounds in concentrations higher than the maximum permitted amount.

## Figures and Tables

**Table 1:** Porphyrin polymeric coatings compositions and deposition details.

Sensor	Monomer Abbreviation	Electropolymerisation Conditions	
		Potential Range, V	Solvent
**1**	CoTrisAminoTPP	−0.3 to 1.3	CH_2_Cl_2_ + 2% ACN
**2**	FeClMonoAminoTPP	−0.5 to 1.4	CH_2_Cl_2_
**3**	MnClMono-AminoTPP	−0.5 to 1.5	CH_2_Cl_2_
**4**	CuTrisAminoTPP	−0.5 to 1.5	CH_2_Cl_2_
**5**	CoTPP-Mono-O-(CH_2_)_5_-pyrr	−0.5 to 1.5	CH_2_Cl_2_
**6**	MnClTPP-Mono-O-(CH_2_)_10_-pyrr	−0.3 to 1.3	CH_2_Cl_2_
**7**	TPP-Mono-O-(CH_2_)_10_-pyrr	−0.3 to 1.4	CH_2_Cl_2_
**8**	TPP-bis-pyrr	−0.3 to 1.4	CH_2_Cl_2_

**Table 2:** The analysed wines. Year of production: 2007.

Wine Code	Grape	Cantina	Name	Geographic Area
PR1	Primitivo, 100%	Soloperto	Rubinum	-
PR2		Varvaglione	Papale primitivo di Manduria	Leporano
PR3		Conti Zecca	Donna Marzia	Leverano
PR4		Tre Pini	Primitivo di Cassano Murge	Cassano Murge
PR5		Castel di Salve	Cento su cento	Tricase
PR6		Cantina coop R.F.	Primitivo	Mesagne
PR7		Racemi	Felline	Manduria
PR8.1		Antica masseria del sigillo	Primo sigillo	Guagnano
PR8.2		Antica masseria del sigillo	Siaillo	Guagnano
PR9		Villa Santerà	Leone de castris	Salice Salentino
PR10		Produttori vini Manduria	Madrigale	Manduria
NA1	Negroamaro, 100%	Conti Zecca	Cantalupi	Leverano
NA2		Mocavero	Negroamaro Salento	Arnesano
NA3		Carrozzo	Carmino	Magliano
NA4		Benegiamo	Filimei l’astore Masseria	Cutrofiano
NA5		Paolo Leo	Orfeo	San Donaci
NA6		Cantele	Lutroc San Chirico	Guagnano
NA7		Vallone	Santi Dimitri Aruca	Calatina
NA8		Cantine due palme	Canonico	Cellino S. Marci
NA9		Calò e figli	Mjere	Tuglie
NA10		Vitic. RIA	Effige	Collepasso
NA11		Vigneti reale	Norie negroamaro	Lecce
NA12		Santa Maria del Morige	Negroamaro	Cellino S. Marco
NA13		Cantina Rosa del Golfo	Scaliere	Alezio
NA14		Cantele (with adulterations)	Lutroc San Chirico	Guagnano

**Table 3:** The amounts of wine defect compounds in artificial wine calibration solutions and in Negroamaro wine (NA14).

Defect Compound	Max. Permitted Amount, mg/L	Alarm Level Amount, mg/L	Defect Level Amount, mg/L	NA14, Range in mg/L
BA	0.015	3	6	3–1000
AA	750	1000	2000	1000–100,000
IA	30	300	600	300–10,000
ME	1	2	4	1–100

**Table 4:** PLS-DA confusion matrix of Primitivo and Negroamaro wines classification obtained by cross validation procedure.

Expected	Predicted	
	PR	NA
**PR**	6	4
**NA**	3	11

**Table 5:** PLS-DA confusion matrix of original Negroamaro wines and wines with adulterations obtained by cross validation procedure.

Expected	Predicted	
	NA	NA + Faults
**NA**	13	1
**NA + faults**	0	15

## Supplementary Materials

The following are available online at http://www.mdpi.com/1424-8220/18/8/2584/s1, Figure S1: The acquired potentiometric data of the developed potentiometric e-Tongue array in artificial wine solutions with different faults compounds: (A) benzaldehyde, BA; (B) acetic acid, AA; (C) isoamyl alcohol, IA; (D) methionol, ME., Figure S2: The potentiometric response of potentiometric e-Tongue in real Negroamaro wine doped with fault compounds.
