# Supplementary material for: Electronic Tongue for Brand Uniformity Control: A Case Study of Apulian Red Wines Recognition and Defects Evaluation
Source: Sensors (Basel). 2018 Aug 7;18(8):2584. doi: 10.3390/s18082584 (PMC6111746; doi:10.3390/s18082584)
Supplement: Supplementary file 1 [file sensors-18-02584-s001.pdf]

Electronic Supporting Information (ESI) for

Electronic tongue for brand uniformity control: a case study of Apulian red wines recognition and defects evaluation

Larisa Lvova [1,2,*], Irina Yaroshenko [1], Dmitry Kirsanov [1,3], Corrado Di Natale [1], Roberto Paolesse [1] and Andrey Legin [1,3]

[1] Laboratory of Artificial Sensory Systems, ITMO University, St. Petersburg, Russian Federation
[2] Department of Chemical Sciences and Technology, University 'Tor Vergata', Rome, Italy
[3] Institute of Chemistry, St. Petersburg State University, St. Petersburg, Russian Federation
* Corresponding author: larisa.lvova@uniroma2.it; Tel.: +39-06-7259-4755

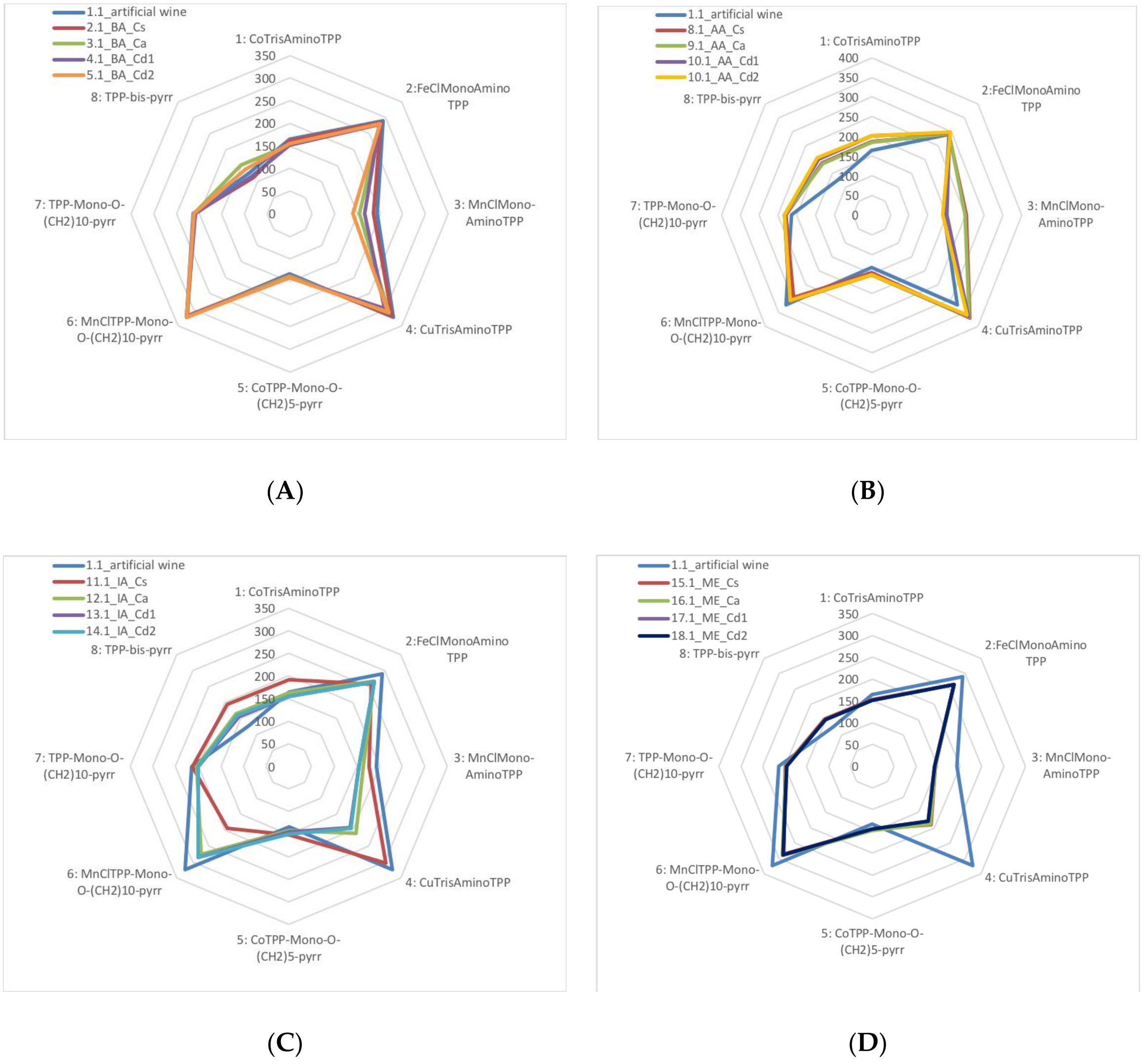

Figure S1. The acquired potentiometric data of the developed potentiometric e-Tongue array in artificial wine solutions with different faults compounds: (A) benzaldehyde, BA; (B) acetic acid, AA; (C) isoamyl alcohol, IA; (D) methionol, ME.

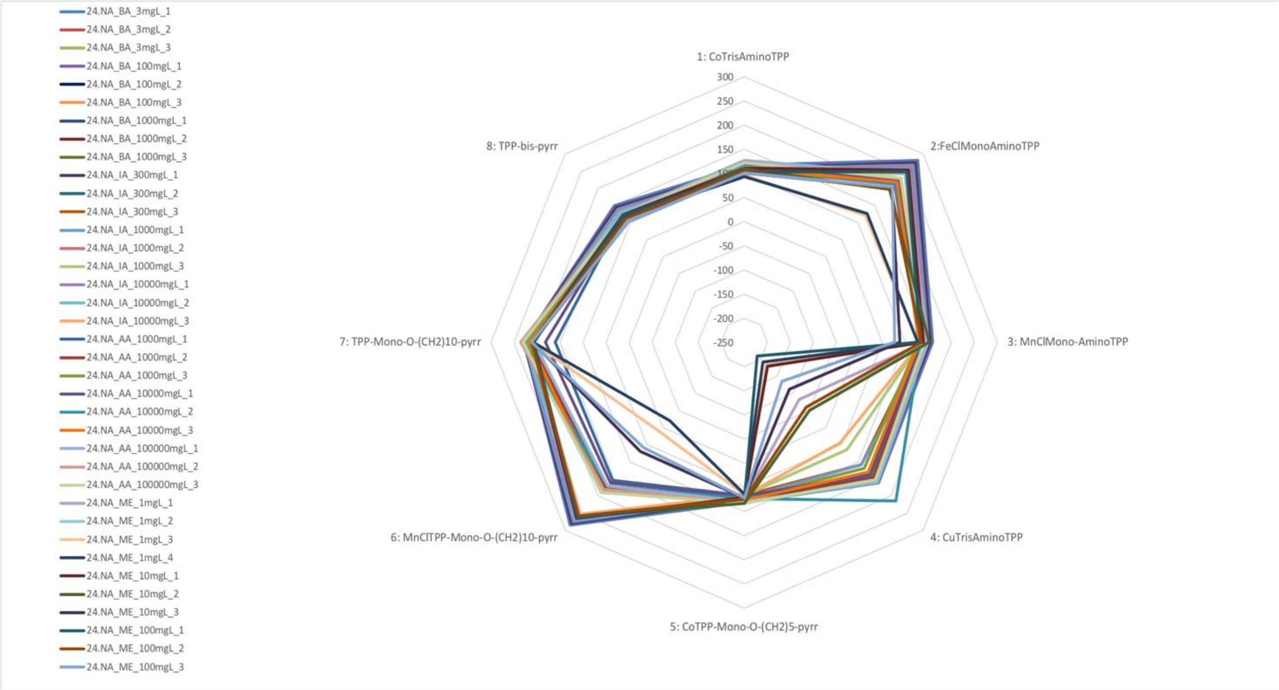

Figure S2. The potentiometric response of potentiometric e-Tongue in real Negroamaro wine doped with fault compounds.
